# DNAzyme-Amplified Label-Free Biosensor for the Simple and Sensitive Detection of Pyrophosphatase

**DOI:** 10.3390/bios11110422

**Published:** 2021-10-28

**Authors:** Cheng-Yu Lee, Chi-Hsiang Liao, Nei-Mei Fang, You-Zung Hsieh

**Affiliations:** 1 Department of Applied Chemistry, National Yang Ming Chiao Tung University, Hsinchu 300, Taiwan; Leec141@mcmaster.ca (C.-Y.L.); qqwwer33@gmail.com (C.-H.L.); alivia520520@gmail.com (N.-M.F.); 2 Center for Emergent Functional Matter Science, National Yang Ming Chiao Tung University, Hsinchu 300, Taiwan

**Keywords:** biosensor, PPase, DNAzyme, ATMND, AP site

## Abstract

The level of pyrophosphatase (PPase) expression has been suggested as a potential biomarker of various cancers, and its prognostic value has been evaluated in patients suffering from lung cancer, colorectal cancer, and hyperthyroidism. However, the detection of PPase usually needs specific materials that require complicated, time-consuming reactions with restricted linear range and sensitivity, limiting their application in early clinical diagnosis. Herein, we developed a DNAzyme-based biosensor for the detection of PPase. In the presence of PPase, pyrophosphate (PPi) and Cu^2+^ ions released from the PPi–Cu^2+^–PPi complex induce the cleavage of the DNAzyme and the corresponding substrate. An apurinic/apyrimidinic (AP) site was elaborately designed within substrates that could encase the fluorophore 2-amino-5,6,7-trimethyl-1,8-naphthyridine (ATMND). The fluorescence of ATMND was initially quenched but restored when the DNAzyme/substrate complex was hydrolyzed with the release of ATMND. In this way, the PPase activity can be estimated by detecting the increased fluorescence of the released ATMND. Under optimized conditions, the activity of PPase could be analyzed at concentrations from 0.5 to 1000 mU, with the lowest detectable concentration being 0.5 mU. This work lays a foundation for developing a DNAzyme-amplified fluorescent biosensor with a high sensitivity, a wide linear range, and single-step operation for use as an easy diagnostic for PPase analysis.

## 1. Introduction

Inorganic pyrophosphatase (PPase) is a hydrolytic enzyme that can specifically catalyze the fragmentation of a molecule of pyrophosphate (PPi) into two separate orthophosphate (Pi) ions. This highly exergonic reaction can help some less-favorable reactions to reach completion [1], thereby playing a significant role in many biological reactions, including DNA, RNA, and protein synthesis; calcium absorption; lipid synthesis; the degradation of biological products [2]. The expression levels of PPase have been suggested as a potential biomarker of various cancers, and their prognostic value has been evaluated in patients suffering from lung cancer, colorectal cancer, and hyperthyroidism [3]. As such, the accurate quantification of PPase is vital for clinical diagnosis and its subsequent management. So far, several analytical techniques have been developed for PPase detection. Wang et al. used a G-quadruplex modified screen-printed electrode for PPase detection [4]. A two-stage reaction of H_2_O_2_–TMB was carried out upon the release of Cu^2+^ from the PPi complex after hydrolysis by PPase. The change in TMB (3,3′,5,5′-tetramethylbenzidine) redox signaling was used to estimate the activity of PPase with a detection limit of 0.6 mU/mL. Han and his team synthesized an artificial peroxidase [Mn_2_(bpmp)]^3+^ complex [5], which can trigger a colorimetric assay using 2,2′-azino-bis(3-ethylbenzothiazoline-6-sulphonic acid) (ABTS) and H_2_O_2_. This property of peroxidase was inhibited by PPi and restored by PPase catalysis, which was used for PPase detection with a detection limit of 0.5 U/mL. Lin et al. combined hyper-branched rolling-circle amplification (HRCA) with a padlock design for PPase detection [6]. The Cu^2+^ released from the PPi complex by PPase hydrolysis was first reduced to Cu^+^ by sodium ascorbate. Then, Cu^+^ catalyzed the cyclization of 5′-azide and 3′-alkyne tagged padlock probes to create the circular template for the HRCA reaction after treatment with Exo I and Exo III. The fluorescence generated by SYBR Green I was used to estimate the PPase activity. Other methods using different designs or strategies that have been reported for PPase are listed in Table S1. In most of these approaches, the specific PPi was chosen as the first reaction substrate based on the native property of PPase and the definition of its activity. Further reactions, from basic oxidation/reduction to powerful DNA polymerizations, were carried out, and the resulting signal was used for PPase activity quantitation. However, these methods need specific materials that require difficult synthesis, complicated procedures for multi-stage reactions, or expensive reagents and instruments. In addition, their linear range is usually narrow and needs further improvement. Although PPase is important for a clinical diagnosis, there is no easy way to measure PPase activity. Therefore, there is significant demand for a simple, cost-effective method with a large linear range and high sensitivity for PPase analysis.

DNAzymes, artificial deoxyribozymes in the form of single-strand DNA (ssDNA), are capable of triggering certain chemical or biological reactions upon binding to their substrates through, for example, hydrogen bonding, π–π interactions, and metal ion coordination [7]. DNAzymes have become popular bioanalytical elements due to their high level of catalytic ability in the cleavage of RNA substrates [8] and their extremely high stability in various environments, making them easy to handle experimentally. Many different types of DNAzymes have been developed, including Mg^2+^-dependent cleavage DNAzyme [7,9], Cu^2+^-dependent cleavage DNAzyme [10,11], Pb^2+^-dependent cleavage DNAzyme [12,13], and histidine-dependent DNAzyme [14,15]. Most DNAzymes require certain metal ions as cofactors to display their catalytic activity. As such, DNAzymes have great potential to function as highly selective biosensors. So far, DNAzymes are not widely used in biosensor development, and most of the designs need the labeling of fluorophores and quenchers first. In this study, we designed a label-free, self-assembled DNAzyme/substrate complex to first approach the detection of PPase, providing an alternative DNAzyme-based analytical method.

The fluorophore–quencher system has been widely used in biosensing fields, especially in target oligonucleotide detection [16,17]. The fluorophore is quenched internally at the beginning and is later restored when recognized and bound to its target molecule. The restoration of fluorescence depends on the structural switching of fluorophore–quencher-labelled oligonucleotides, which may interfere with the interactions between DNAs and their target molecules [18,19,20]. Each target requires a unique fluorophore-labelled oligonucleotide, which has a complicated labelling and purifying process, so its cost is far higher than that of regular oligonucleotides. Therefore, label-free fluorescent sensors are attractive due to their ease of operation and likely increased sensitivity due to less potential interference with the interactions of DNA [21,22,23,24]. In our previous works, a label-free photocurrent biosensor for pyrophosphatase analysis was developed [25]. With the assistance of a copper complex, a polymerization reaction was triggered on a quantum-dots-modified indium-tin oxide (ITO) electrode in the presence of PPase, allowing for an efficient and simple way to determine the PPase activity on disposable electrodes. However, the fabrication of the electrode needs well-trained specialists, and the quantum dots are not environmentally friendly. Finding a more generic method to estimate PPase activity was the goal of this project.

**Scheme 1:**
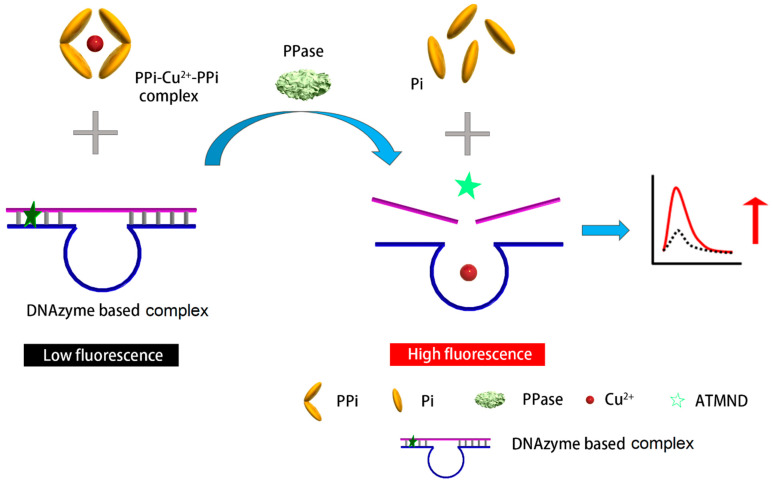
Schematic representation of the designed substrate/DNAzyme-based biosensor for the fluorescent detection of PPase.

In this study, we developed a label-free fluorescent method for the simple and cost-effective detection of PPase activity with a large linear range and high sensitivity, as illustrated in Scheme 1. In the absence of PPase, the PPi–Cu^2+^–PPi complex was stable and unaffected. When PPase was present in the system, however, each PPi moiety in the PPi–Cu^2+^–PPi complex would fragment into two Pi units, leading to the release of Cu^2+^. The Cu^2+^ was the cofactor used to trigger the DNAzyme cleavage reaction. Here, we elaborately introduced an AP site into the substrate of a DNAzyme. The AP site is a location in DNA (and less likely in RNA) that has no nitrogenous base and is formed spontaneously or as a result of DNA damage [26]. The AP site trapped the fluorophore 2-amino-5,6,7-trimethyl-1,8-naphthyridine (ATMND) so that ATMND, as displayed in Figure S1, was embedded in the substrate/DNAzyme pair [27]. The aforementioned interaction released Cu^2+^, which then triggered the DNAzyme to break its substrate into two parts, thereby releasing ATMND and elevating the fluorescent signal. Different from previously reported PPase detection methods, this new method required no labelling processes, expensive enzymes and reagents, or complicated synthesis and multiple reactions. The major advantage is a simplified operation protocol with stable PPi–Cu^2+^ and sensitive DNAzyme–ATMND complexes. Only one mixing step is required before measuring, so generic laboratories could easily utilize it for PPase detection with a high sensitivity, wide linear range, and low cost.

## 2. Materials and Methods

### 2.1. Chemicals and Reagents

All chemicals were of reagent-grade or higher. All stock and working solutions were prepared using Milli-Q deionized (DI) water (Millipore, Bedford, MA, USA) with a measured resistance of 18.2 MΩ·cm. Copper chloride, 2-amino-5,6,7-trimethyl-1,8-naphthyridine (ATMND), sodium pyrophosphate decahydrate, inorganic pyrophosphatase (PPase), human serum albumin (HSA), and 4-(2-hydroxyethyl)-1-piperazineethanesulfonic acid (HEPES) were purchased from Sigma–Aldrich (St. Louis, MO, USA). Potassium chloride and sodium hydroxide were acquired from J. T. Baker (Phillipsburg, USA). Agarose and gel electrophoresis TAE buffer (including Tris base, acetic acid, and EDTA) were purchased from Bersing Technology (Hsinchu, Taiwan). Two DNA sequences (DNAzyme: 5′-CAA AAG AAT TTT TTC TTT CTC CGG GTC CGG CCC GG-3′; AP site DNA: 5′-CCG _GC CGG CAT AAT CTT TCT TCG A-3′) were obtained from MDBio (Taipei, Taiwan).

### 2.2. Apparatus

Fluorescence spectra were recorded using an F-7000 fluorescence spectrometer (Hitachi, Tokyo, Japan). Gel electrophoresis was performed using a Mini-300 power supply and an MT-108 electrophoresis chamber (Major Science, New Taipei, Taiwan).

### 2.3. DNAzyme-Based Biosensor

The DNAzyme-based biosensor was prepared by mixing the DNAzyme (1 μM, 18 μL) and its substrate (1 μM, 15 μL) in a HEPES buffer solution (10 mM, pH 7.2, 447 μL) containing 1.5 mM of NaCl, heating the mixture at 80 °C for 2 min, adding ATMND (1 μM, 15 μL), and then cooling to 4 °C.

### 2.4. Detection of Ppase Activity

For the analysis of the PPase activity, a detection solution containing PPi–Cu^2+^–PPi was prepared by mixing PPi and copper chloride. Prior to the detection of PPase activity, the sample solution (1 μL) was mixed with the PPi–Cu^2+^–PPi detection solution (4 μL), and then the DNAzyme-based biosensor solution (495 μL) was added. The final concentration of the DNAzyme complex was 30 nM. The mixture was left standing for 4 h at 25 °C; then, its fluorescence spectrum was measured via the fluorescence spectrometer (excitation wavelength: 360 nm).

## 3. Results and Discussion

### 3.1. Characterization of the DNAzyme and Its Substrate

**Figure 1:**
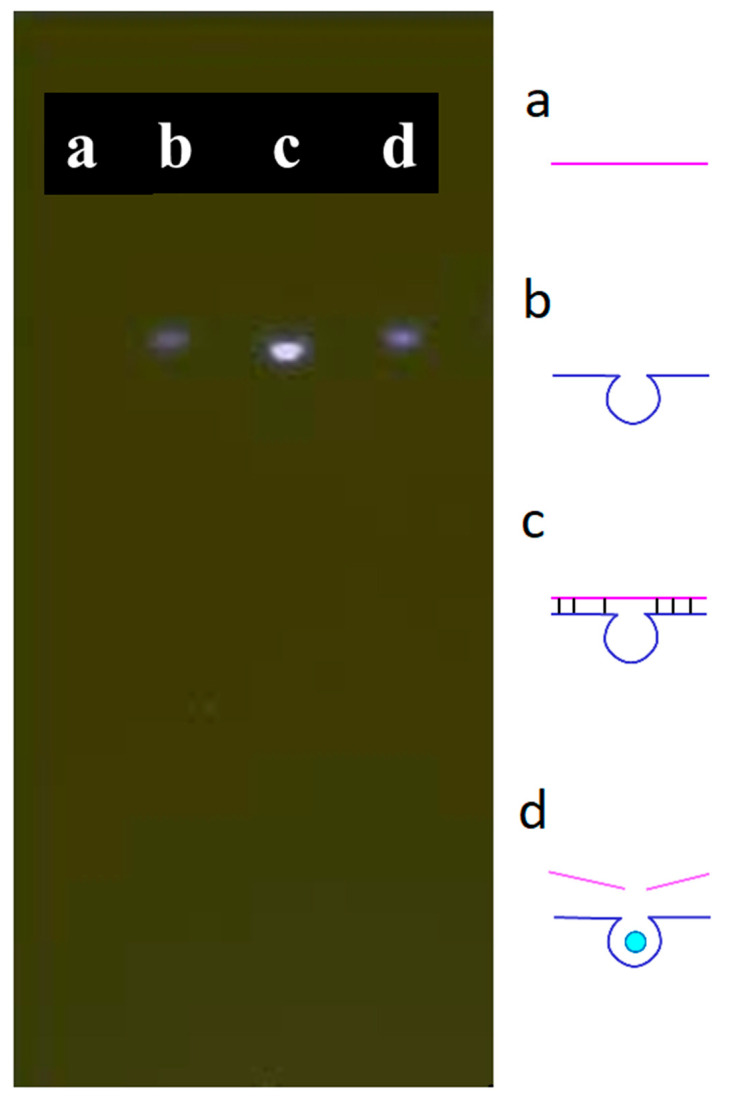
Gel electrophoresis of the designed substrate and DNAzyme: (**a**) substrate, (**b**) DNAzyme, (**c**) substrate + DNAzyme, and (**d**) substrate + DNAzyme + Cu^2+^. Conditions: 500 nM for each compound, 50 nM of Cu^2+^. Separated with 2% agarose gel with added 1x SYBR^®^ Green I; 300 V for 10 min.

A substrate featuring an AP site was hybridized with the DNAzyme [28] to form a complex. The AP site was located at the fourth base from the 5′ end of the substrate; its complex could trap ATMND and quench its fluorescence. If the double-stranded form of the complex were to become denatured, i.e., after adding Cu^2+^, ATMND would be released, thereby re-emitting its fluorescence because the substrate itself could not trap ATMND in its single-stranded form. Verifying the behavior of the designed substrate and DNAzyme, Figure 1 presents the results of the gel electrophoresis of the complex in the absence and presence of Cu^2+^. Briefly, the substrate and DNAzyme were loaded into channels a and b, respectively. No significant band appeared in channel a due to the low fluorescent response of ssDNA towards SYBR Green I. Some intramolecular hybridization occurred within the DNAzyme sequence, as evidenced by a weak fluorescent band in channel b. After mixing both the substrate and the DNAzyme in channel c, a significant bright band appeared, signifying that the hybridization of the two substances had occurred. This complex was able to capture and quench the fluorophore ATMND before analysis. In channel d, the brightness of this band decreased after adding Cu^2+^, which triggered the cleavage of the substrate by the DNAzyme and denatured the double-stranded form of the complex. The dissociated DNAzyme and substrate lost their quenching property to ATMND. These findings indicate that the substrate and the DNAzyme functioned as expected in the absence and presence of Cu^2+^.

**Figure 2:**
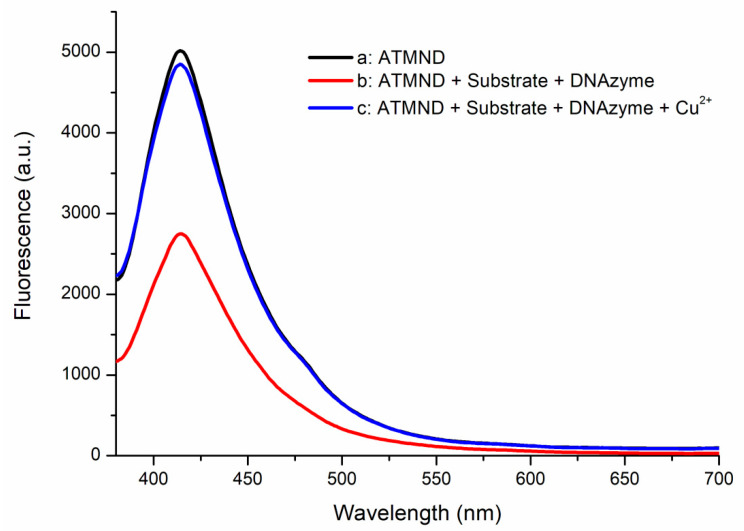
Fluorescence spectra of a: ATMND; b: ATMND with the substrate and DNAzyme; and c: ATMND with the substrate, DNAzyme, and Cu^2+^. Conditions: 30 nM of the DNAzyme complex (30 nM of ATMND, 30 nM of the substrate, and 33 nM of the DNAzyme), 10 nM of CuCl_2_.

The performance of the designed substrate and DNAzyme was also examined using fluorescence spectrometry. Figure 2 reveals that ATMND displayed a significant fluorescence signal at 415 nm (curve a). The intensity of this emission signal decreased greatly after both the substrate and the DNAzyme were added (curve b)—the result of the fluorescence quenching of ATMND when the combined substrate and DNAzyme formed their complex [29,30]. When the solution used to obtain curve b was treated with 10 nM of Cu^2+^ to activate the DNAzyme, the intensity of the fluorescence signal at 415 nm was restored (curve c). The trapped ATMND was released after the DNAzyme cleaved its substrate, hydrolyzing the complex and restoring the fluorescence of ATMND. These spectral observations confirmed the functional performance of the designed substrate and DNAzyme system in the absence and presence of Cu^2+^ ions, encouraging its application in the proposed detection method. For further information, the relationship between Cu^2+^ concentration and restored fluorescence is shown in Figure S2. We mixed the DNAzyme–substrate with the quenched ATMND complex (final concentration of the DNAzyme complex at 30 nM) and Cu^2+^ ions in various concentrations and monitored the change in fluorescence over a certain time period. A linear relationship was observed between the fluorescence change and Cu^2+^ concentrations in a logarithmic form from 1 nM to 1 μM. The obtained results show that the DNAzyme–substrate complex is sensitive to Cu^2+^ ions, even at a concentration as low as 1 nM. Moreover, the linear trend indicates that the cleavage rate of the designed DNAzyme–substrate complex is related to Cu^2+^ ions, which are released from PPi–Cu^2+^–PPi by PPase digestion. Given that PPase digestion is a zero-order reaction, Cu^2+^ ions are released at a rate corresponding to the PPase activity. Therefore, the restoration of ATMND fluorescence could be used to estimate PPase activity.

### 3.2. Optimization of the Biosensor

**Figure 3:**
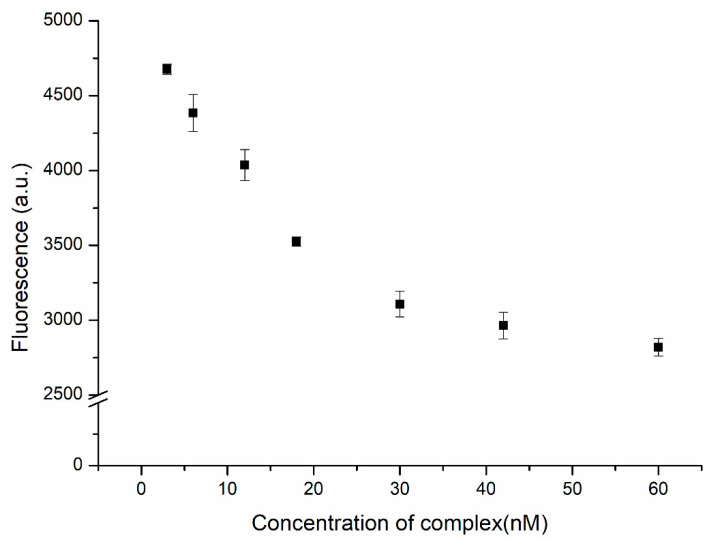
Fluorescence intensity of ATMND (30 nM) in the presence of the DNAzyme complex (3, 6, 12, 18, 30, 42, and 60 nM).

We used the complex formed from the substrate and the DNAzyme as the quencher of ATMND. In the presence of Cu^2+^ ions, ATMND was released as a result of the hydrolysis of this complex. In the absence of the quenching effect of the complex, the fluorescence of ATMND was restored. A better performance of this system could be achieved at a higher signal-to-noise (S/N) ratio. An excess of ATMND would result in not all of the fluorescence being quenched by the complex prior to detection, leading to strong background noise. In contrast, using an excess amount of the complex might lead to the proposed biosensor having blind areas, i.e., no response to the presence of the analyte, especially at low concentrations of the analyte. Therefore, the amount of complex required for the quenching of ATMND would need to be as high as possible to provide the lowest background noise, but an adequate response to the analyte would also be necessary. Figure 3 displays the fluorescence intensity of ATMND (at 30 nM) plotted with respect to the concentration of the complex. The fluorescence intensity decreased upon increasing the concentration of the complex from 3 to 30 nM and approached an asymptote thereafter, indicating that the complex had trapped most of the ATMND. The saturation ratio between ATMND and the complex was close to 1, consistent with the values given in previous reports [31,32]. As a result, a concentration of 30 nM for the complex was used as the optimized value for subsequent experiments.

**Figure 4:**
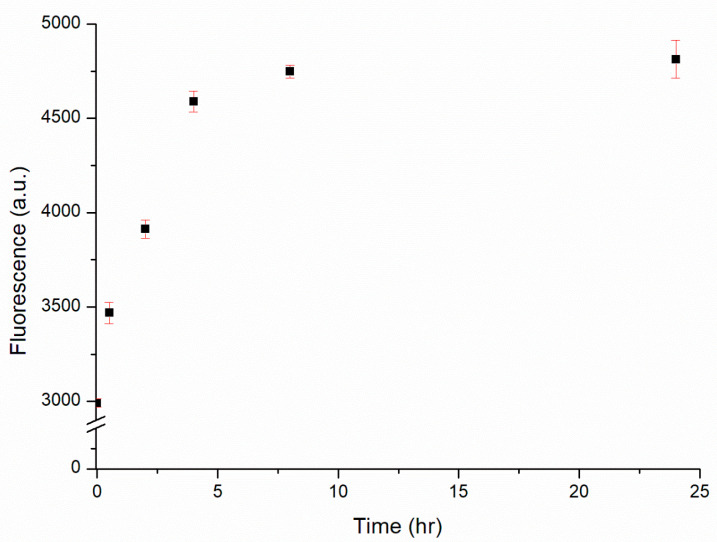
Fluorescence intensity of the DNAzyme complex (30 nM) in the presence of 1 μM Cu^2+^ over reaction times from 0.5 to 24 h.

The designed substrate was comprised a substrate sequence featuring one AP site and a related DNAzyme sequence. The hydrolysis activity of this DNAzyme was triggered by the presence of Cu^2+^ ions. Specifically, the presence of Cu^2+^ ions induced the hydrolysis of the substrate into two residue sequences that denatured the complex into the form of ssDNA. Thereafter, each Cu^2+^ ion could trigger the hydrolysis of another complex, repeating this process until all substrates were hydrolyzed. To ensure the maximal sensitivity of the proposed biosensor, the reaction time was optimized. Figure 4 displays the relationship between the fluorescence intensity and the reaction time. The fluorescence intensity increased upon extending the reaction time, indicating that the hydrolysis of the substrate and the release of ATMND were continuing processes. Nevertheless, the enhancement in fluorescence leveled off after 4 h, suggesting that the hydrolysis of the substrate was approaching completion and that the reaction time was optimized. Extending the reaction time thereafter increased the fluorescence intensity only slightly. Therefore, a reaction time of 4 h was chosen for the subsequent experiments.

**Figure 5:**
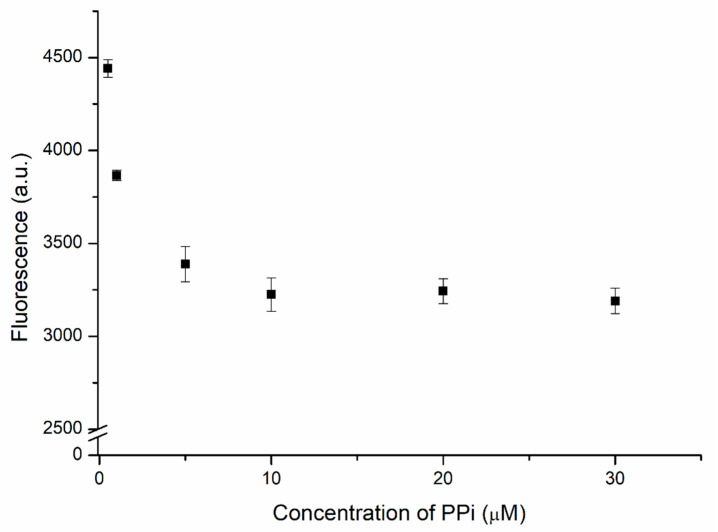
Fluorescence intensity of the DNAzyme complex (30 nM) in the presence of 1 μM of Cu^2+^ and PPi at various concentrations.

The proposed biosensor used PPi-captured Cu^2+^ as a trigger for the hydrolysis of the designed complex. Therefore, the concentration of PPi required optimization to ensure top performance. Two units of PPi, which chelates the Cu^2+^ ion, is the substrate of PPase. The DNAzyme complex was added after the mixing of 1 μM of Cu^2+^ with various PPi concentrations. Then, the solution was incubated for 4 h before measuring. Figure 5 displays the fluorescence intensity of the biosensor measured at various concentrations of PPi. Strong fluorescence appeared at low PPi concentrations. This intensity decreased significantly upon increasing the concentration of PPi, reflecting the greater chelation of Cu^2+^, which left a lower concentration of free Cu^2+^ ions to trigger the hydrolysis of the substrate by the DNAzyme in the absence of PPase. When 10 μM of PPi had been added, the fluorescence intensity became steady; even when the concentration of PPi reached 30 μM, there was no significant decrease in fluorescence, suggesting that the chelation of Cu^2+^ by PPi had reached a stable situation. Because a large concentration of PPi would lower the sensitivity of the biosensor, the optimized concentration of PPi used to capture the Cu^2+^ ions in subsequent experiments was 10 μM, thereby ensuring that no free Cu^2+^ ions would be available to trigger the DNAzyme-mediated hydrolysis prior to the catalysis initiated by PPase.

### 3.3. Sensitivity and Recovery of the DNAzyme-Based PPase Biosensor

**Figure 6:**
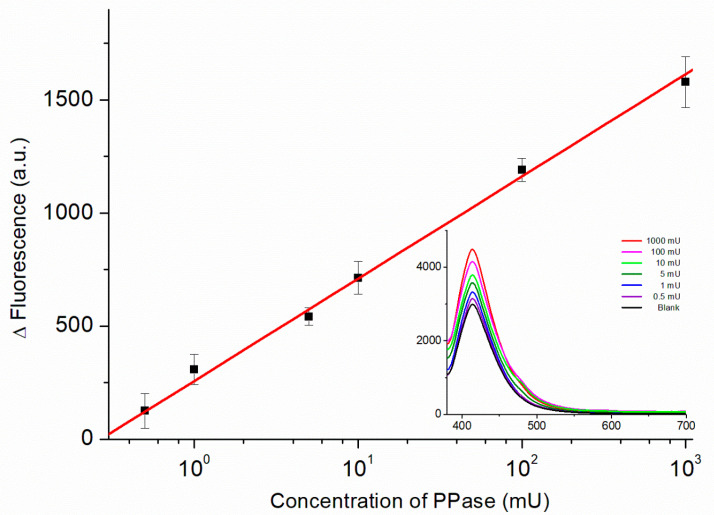
Calibration curve of the PPase biosensor for PPase concentrations from 0.5 to 1000 mU (logarithmic scale). Inset: Fluorescence spectra of the biosensor recorded in the presence of PPase at various concentrations used to obtain the calibration curve.

Figure 6 displays the calibration curve obtained from the fluorescence spectra of the biosensor recorded in the presence of PPase at various concentrations. The relationship between the fluorescence intensity (ΔF = F_PPase_ − F_Blank_) and the PPase activity (on a logarithmic scale) was linear from 0.5 to 1000 mU using the regression equation:

(1) ΔF=451.8 log[PPase]+258.0

The values of 28.1 and 35.8 act as uncertainties for slope and intercept, respectively, and a correlation of determination (*R*^2^) of 0.99 was used. The lowest concentration point was 0.5 mU, which shows a comparable performance with previously reported PPase biosensors (Table S1). Moreover, in comparison to our previous works [25], this method is more sensitive with a one-step procedure. The use of fluorescent analysis also makes the proposed method feasible in most laboratories that measure PPase activity.

Finally, the practical applicability of this PPase biosensor was studied using human serum albumin (HSA), a major component of human blood, as the matrix. Various concentrations of PPase were spiked into a solution of the biosensor in HSA (50 mg/L), and the resulting fluorescence intensities were substituted into the calibration curve to determine the recoveries. In the presence of 10, 100, and 1000 mU of spiked PPase, the estimated recoveries were 97.9 ± 1.94, 95.7 ± 1.38, and 96.6 ± 1.90%, respectively. Thus, the performance of this PPase biosensor appears suitable for routine analysis with a good reproducibility (RSDs: <1.94%).

## 4. Conclusions

In summary, we developed a simple and label-free fluorescent biosensor for the detection of PPase utilizing Cu^2+^-dependent DNAzyme-mediated cleavage. In the presence of PPase, Cu^2+^ ions are released from the PPi–Cu^2+^–PPi complex; these Cu^2+^ ions then trigger DNAzyme to cleave its substrate, resulting in fluorescent enhancement through the release of ATMND. Compared with previous biosensors used for the detection of PPase that need multi-step reactions, expensive enzymes and reagents, or complicated synthesis procedures, the DNAzyme-based biosensor can accomplish its goal within a short span of time with a good stability. The sensing system exhibited a good linearity in terms of the fluorescent response towards the PPase concentration, with the lowest concentration of PPase being 0.5 mU. This biosensor was applied to the detection of PPase in a human serum albumin matrix with satisfactory results. This DNAzyme-based approach provides an effective strategy for developing a biosensor with a high sensitivity and a wide linear range in the detection of PPase, potentially allowing the simple and accurate detection of PPase in clinical samples for the early diagnosis of various diseases.

## Supplementary Materials

The following are available online at https://www.mdpi.com/article/10.3390/bios11110422/s1, Figure S1: The chemical structure of ATMND, Figure S2: Fluorescence intensity of the DNAzyme complex in the presence of Cu^2+^ in the range from 1 to 1000 nM (logarithmic scale), Table S1: Reported PPase biosensors in recent years.
