# Supplementary material for: DNAzyme-Amplified Label-Free Biosensor for the Simple and Sensitive Detection of Pyrophosphatase
Source: Biosensors (Basel). 2021 Oct 28;11(11):422. doi: 10.3390/bios11110422 (PMC8615721; doi:10.3390/bios11110422)
Supplement: Supplementary file 1 [file biosensors-11-00422-s001.zip › biosensors-1376163-SI.pdf]

Table S1 Reported PPase biosensors in recent years.

Detection method	Detection range (mU/mL)	LOD (mU)	Assay time	Ref
Fluorescence	1 – 20	1.0	44 h + 30 min	[1]
Fluorescence	1 – 200	1.0	60 min[2]	[2]
Fluorescence	0.05 – 25	0.02	250 min	[3]
Colorimetric	25 – 400	10	30 min[3]	[4]
Colorimetric	30 – 400	10	30 min	[5]
Colorimetric	0 – 8000	0.5	3 days + 130 min	[6]
Electrochemistry	1.0 – 50	0.6	315 min	[7]
Photocurrent	0.8 – 5000	0.41	310 min + 120 min	[8]
Fluorescence	0.5 – 1000	0.50[1]	242 min	this work

[1] Lowest detectable concentration
[2] Synthesis of GQD is not included
[3] Synthesis of Au NP is not included

[1] J. Sun, F. Yang, D. Zhao, X. Yang, Highly Sensitive Real-Time Assay of Inorganic Pyrophosphatase Activity Based on the Fluorescent Gold Nanoclusters, Anal. Chem. 86(15) (2014) 7883-7889.

[2] X. Zhu, J. Liu, H. Peng, J. Jiang, R. Yu, A novel fluorescence assay for inorganic pyrophosphatase based on modulated aggregation of graphene quantum dots, Analyst 141(1) (2016) 251-255.

[3] Y. Zhang, Y. Guo, M. Zhao, C. Lin, Z. Lin, F. Luo, G. Chen, Fluorescence biosensor for inorganic pyrophosphatase activity, Anal. Bioanal. Chem. 409(4) (2017) 999-1005.

[4] J. Deng, Q. Jiang, Y. Wang, L. Yang, P. Yu, L. Mao, Real-Time Colorimetric Assay of Inorganic Pyrophosphatase Activity Based on Reversibly Competitive Coordination of Cu^{2+} between Cysteine and Pyrophosphate Ion, Anal. Chem. 85(19) (2013) 9409-9415.

[5] L. Zhang, M. Li, Y. Qin, Z. Chu, S. Zhao, A convenient label free colorimetric assay for pyrophosphatase activity based on a pyrophosphate-inhibited Cu^{2+}–ABTS–H_2O_2 reaction, Analyst 139(23) (2014) 6298-6303.

[6] Y. Lee, S. Yoo, S. Kang, S. Hong, M.S. Han, An $[Mn_2(bpmp)]^{3+}$ complex as an artificial peroxidase and its applications in colorimetric pyrophosphate sensing and cascade-type pyrophosphatase assay, Analyst 143(8) (2018) 1780-1785.

[7] Y. Wang, Y. Wu, W. Liu, L. Chu, Z. Liao, W. Guo, G.-Q. Liu, X. He, K. Wang, Electrochemical strategy for pyrophosphatase detection Based on the peroxidase-like activity of G-quadruplex-Cu^{2+} DNAzyme, Talanta 178 (2018) 491-497.

[8] C.-Y. Lee, C.-H. Liao, J.-T. Tso, Y.-Z. Hsieh, A pyrophosphatase biosensor with photocurrent analysis, Sens. Actuators B Chem. 284 (2019) 159-163.

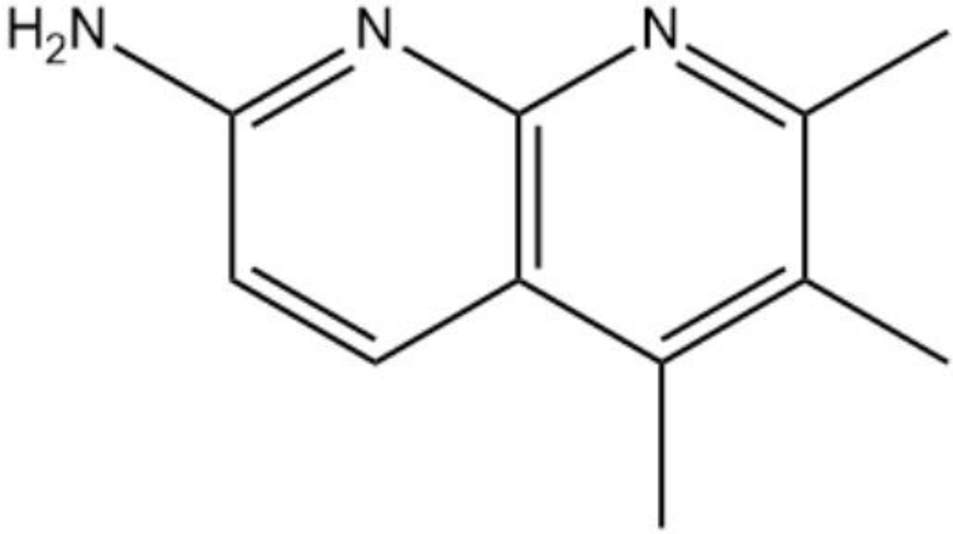

Figure S1. The chemical structure of ATMN..D

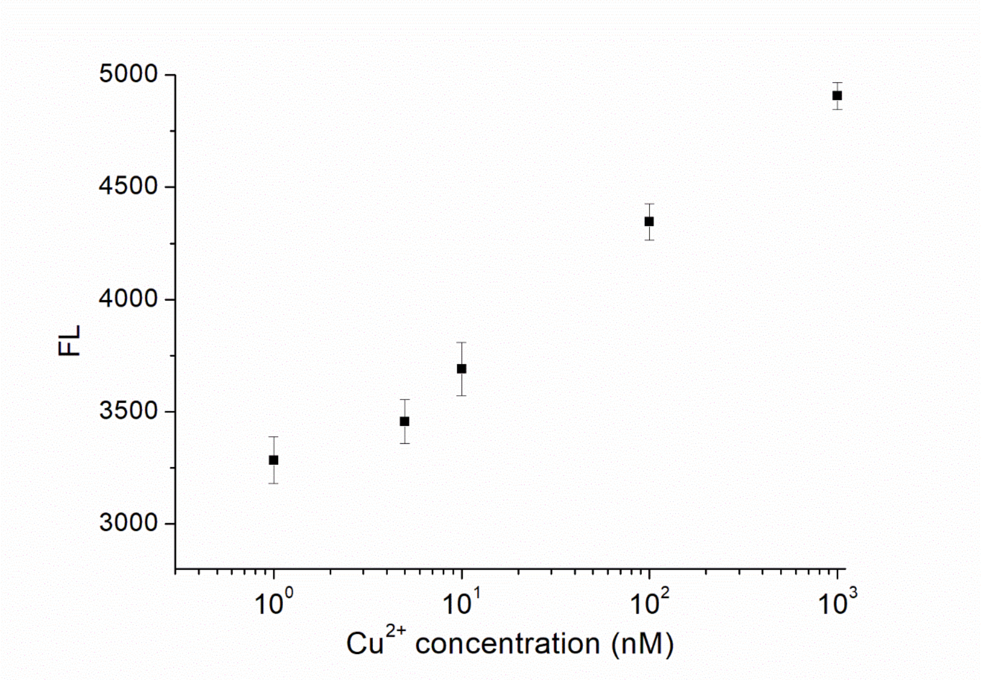

Figure S2. Fluorescence intensity of the DNAzyme complex in the presence of Cu^{2+} in the range from 1 to 1000 nM (logarithmic scale).
